# Electrochemotherapy Causes Caspase-Independent Necrotic-Like Death in Pancreatic Cancer Cells

**DOI:** 10.3390/cancers11081177

**Published:** 2019-08-14

**Authors:** Philana Fernandes, Tracey R. O’Donovan, Sharon L. McKenna, Patrick F. Forde

**Affiliations:** Cancer Research at UCC, Western Gateway Building, University College Cork, T12 XF62 Cork, Ireland

**Keywords:** ECT, necroptosis, apoptosis, ICD, necrostatin-1

## Abstract

Pancreatic cancer represents a major challenge in oncology. Poor permeability of the pancreas and resistance to currently available therapies are impediments to improved patient survival. By transiently increasing cell membrane porosity and increasing drug uptake, Electrochemotherapy (ECT) has the potential to overcome these issues. In this study, we have evaluated the response of human and murine pancreatic cancer cells, in vitro, to electroporation in combination with Bleomycin, Cisplatin, or Oxaliplatin (ECT). The cytotoxic actions of all three drugs are potentiated when combined with electroporation in these cells. The biochemical and morphological changes post ECT are associated with immunogenic cell death that occurs with necroptosis rather than apoptosis. Moreover, ECT-induced cell death is rescued by Nec-1 suggesting that necroptosis may play a role in cell death mediated by cancer therapies.

## 1. Introduction

Pancreatic cancer (PC) is the fourth leading cause of cancer-related death with only a 6% survival at five years. There are few effective therapeutic options available to improve patient survival [1,2]. Surgery still remains the only treatment modality with the potential for cure but due to the generally late diagnoses, more than 80% of these cancers are inoperable [3]. For patients with these tumours, palliative chemotherapy regimens are offered. Unfortunately, chemotherapy offers marginal survival benefits due to the high chemoresistance of pancreatic cancer [4]. Few therapies have shown efficacy in the past and even standard of care therapies, like Gemcitabine (GEM) yield only modest improvements in the mortality of patients with advanced or metastatic disease.

Investigations into improving the use of chemotherapies in pancreatic cancer have centred on improving penetration into the tissue past the dense fibrotic stroma. Additionally, efforts have been focussed on overcoming chemotherapy resistance which tends to occur rapidly following treatment initiation. Electroporation is a non-invasive, non-thermal ablation method used to overcome the barrier of the cell membranes by applying a short and intense electric field creating pores therein [5]. Current electroporation-based therapies for pancreatic cancer in the clinic are based around Irreversible Electroporation (IRE) alone or in combination with chemotherapies and have shown promising data [6]. However, only those patients with locally advanced cancer are eligible and must have demonstrable fitness to tolerate the procedure itself or the accompanying high doses of chemotherapy [7]. Electrochemotherapy (ECT), by contrast, utilizes lower voltages of pulses which is not only safer for the patient but also reduces side effects including damage to surrounding healthy tissue. Furthermore, ECT allows the usage of far lower doses of systemic chemotherapy than IRE as a locoregional increase in drug concentration occurs only at the treatment site.

The type of cell death elicited by therapy and the method by which it is achieved has been shown to be crucial to patient response. Despite ECT being used as a local treatment for skin metastases for over 2 decades now [8], there have been few investigations into the type of cell death elicited by such therapy. PDAC is notoriously resistant to the most well characterized type of cell death, apoptosis, often exhibiting impaired initiation of death receptor initiation by well-known inducers of apoptosis such as the engagement of TNFα, FasL, and TRAIL receptors [9]. PDAC cells can also protect themselves from activating the mitochondrial pathway of apoptosis by overexpressing Bcl-family proteins [9]. Furthermore, PDAC cells have evolved mechanisms to prevent the activation of a specific group of cysteine proteases known as caspases, integral to the apoptotic process, by epigenetically downregulating procaspase gene expression [10]. Despite being the standard of care, treatment with Gemcitabine is very commonly associated with resistance. Thus, there is an urgent need to develop approaches to either overcome apoptosis resistance or to trigger non-apoptotic forms of programmed cell death in PDAC. Necroptosis is a caspase-independent form of regulated cell death that is induced by specific stimuli and the promotion of this, alternative mode of cellular demise, has been shown to be promising in situations where the apoptotic machinery is compromised [11]. Moreover, necrosis and its regulated counterpart, necroptosis, are more often associated with immunogenic cell death (ICD) [12]. This type of death, defined as one that is capable of inducing an adaptive immune response in an immunocompetent host, has important and potentially beneficial consequences for cancer patients, by harnessing their own immune system to target malignant cells.

Given that several lines of evidence suggest that certain therapies are capable of inducing this type of regulated necrosis and components of the necrosome are highly expressed in PDAC [13], this study is an initial investigation into the way in which pancreatic cancer cells die as a result of delivering chemotherapy using electroporation.

## 2. Results

### 2.1. Electroporation in the Presence of Bleomycin, Cisplatin and Oxaliplatin Negatively Affects the Ability of Pancreatic Cells to Recover and Proliferate

We initially examined the ability of human (PANC-1) and murine (Pan02) pancreatic cancer cell lines to recover and form colonies following reversible electroporation. Electroporation parameters were pre-optimised for both cell lines such that high permeabilization was achieved, with marginal cell death in EP buffer (data not shown).

Bleomycin, Cisplatin and Oxaliplatin were used in combination with electroporation as these are the standard drugs accepted in the European Standard Operating Procedures on Electrochemotherapy (ESOPE) protocol [14]. Bleomycin was one of the very first clinically approved drugs to be delivered with electroporation, with intracellular concentrations increasing by as much as 1000-fold in electrochemotherapy treated cells [15]. It is now one of the most common chemotherapies used in combination with electroporation in the clinic.

**Figure 1:**
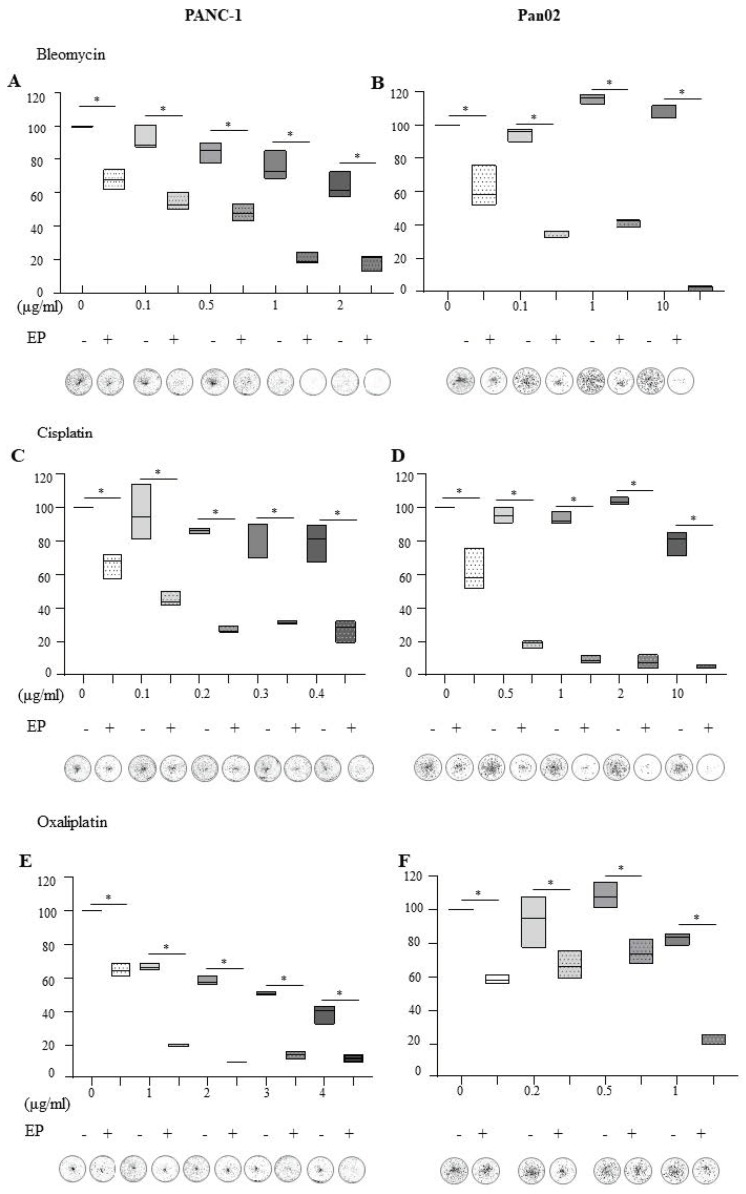
ECT negatively affects the ability of pancreatic cancer cells to recover relative to treatment with chemotherapy alone. (1 million cells were resuspended in EP buffer in the presence or absence of (**A**,**B**, 0.1–10 μg/mL Bleomycin), (**C**,**D**, 0.1–10 μg/mL Cisplatin), or (**E**,**F**, 0.2–4 μg/mL Oxaliplatin). Cells were electroporated and 24 h later, 1000 cells (PANC-1) or 400 cells (Pan02) were seeded in triplicate in complete media in six well plates. The recovery of cells post treatment was quantified by fluorescent intensities of colonies formed after 10–14 days. Each well is a representative of at least nine similar wells (three independent experiments). The data (minimum to maximum) is also presented as floating bar graphs—with the median integrated intensity relative to EP buffer alone denoted with a line. * statistically significant differences in the number of colonies formed when cells were allowed to recover, * *p* = 0.05.

Exposure of PANC-1 cells to increasing concentrations of Bleomycin on its own resulted in a dose-dependent decrease in recovery (Figure 1A). Combining Bleomycin with electroporation significantly impaired the recovery of PANC-1 cells at all doses used (*p* = 0.05) (Figure 1A). Although Pan02 cells were treated with higher doses of Bleomycin alone, there was very little effect on recovery (Figure 1B). Yet, like PANC-1 cells, Bleomycin ECT significantly impaired the recovery of Pan02 cells at all doses (*p* = 0.05).

Low dose Cisplatin (0.1–0.4 μg/mL) did not impact the recovery of PANC-1 cells (Figure 1C). However, electroporation of PANC-1 cells in the presence of all doses of Cisplatin significantly impaired their survival and recovery (Figure 1C) (*p* = 0.05). Pan02 cells were treated with higher doses of Cisplatin yet their recovery remained unaffected (Figure 1D). When combined with electroporation, however, the recovery of Cisplatin-treated cells at all doses was significantly decreased (*p* = 0.05).

Oxaliplatin alone elicited a dose-dependent decrease in PANC-1 recovery (Figure 1E). Oxaliplatin ECT however, significantly affected recovery at all doses (*p* = 0.05). Pan02 cells exhibited no decline in recovery upon Oxaliplatin treatment. However, combination with electroporation led to a significant decline in recovery at 0.5 and 1 μg/mL (Figure 1F) (*p* = 0.05).

These data suggest that PANC-1 and Pan02 cells exhibit different sensitivities to the chemotherapies in terms of their ability to recover following treatment, with Pan02 exhibiting resistance to passive treatment with Bleomycin and Cisplatin. ECT potentiates the chemotoxic effects of these clinically relevant agents, thereby reducing the ability of pancreatic cancer cells to recover from treatment. Concentrations of chemotherapies were chosen for further study based on viability 24 h post-treatment (Supplementary Figure S1) and recovery following ECT.

### 2.2. ECT of Pancreatic Cells Leads to Altered Necrotic-Like Morphology Relative to Drug Alone

A cell succumbing to apoptosis typically exhibits several characteristic morphological features beginning with a profound rearrangement of the nucleus. This entails the initial marginalisation of chromatin followed by its compaction towards the nuclear periphery [16,17]. DNA is then degraded by caspase-activated DNase leading to fragmentation of the nuclear material. The membrane may bleb and the cell may release apoptotic bodies. Thus, the plasma membrane and organelles contained within remain unchanged in a cell undergoing apoptosis until the very late stages of the process. By contrast, cells undergoing necrosis are characterized by an early increase in cellular volume accompanied by an increasingly translucent cytoplasm, finally culminating in a loss of plasma membrane integrity [17]. In some cells undergoing necrosis, the chromatin may become more condensed whilst in others it remains diffuse. The mechanism of cell death following ECT is currently unclear. Electroporation itself has been shown to cause cell swelling and membrane blebbing in the minutes immediately following treatment [18], in addition, a patchwork effect of cell fragments and live fused cells, some multinucleated, has also been observed 24 h post electroporation in fibroblastic cells [19]. Endothelial cells undergoing Bleomycin ECT display a decrease in turgidity, shrink and become spindle-like in the hours subsequent to treatment [20]. These reports suggest that the effect of electroporation itself is likely to be cell-specific.

**Figure 2:**
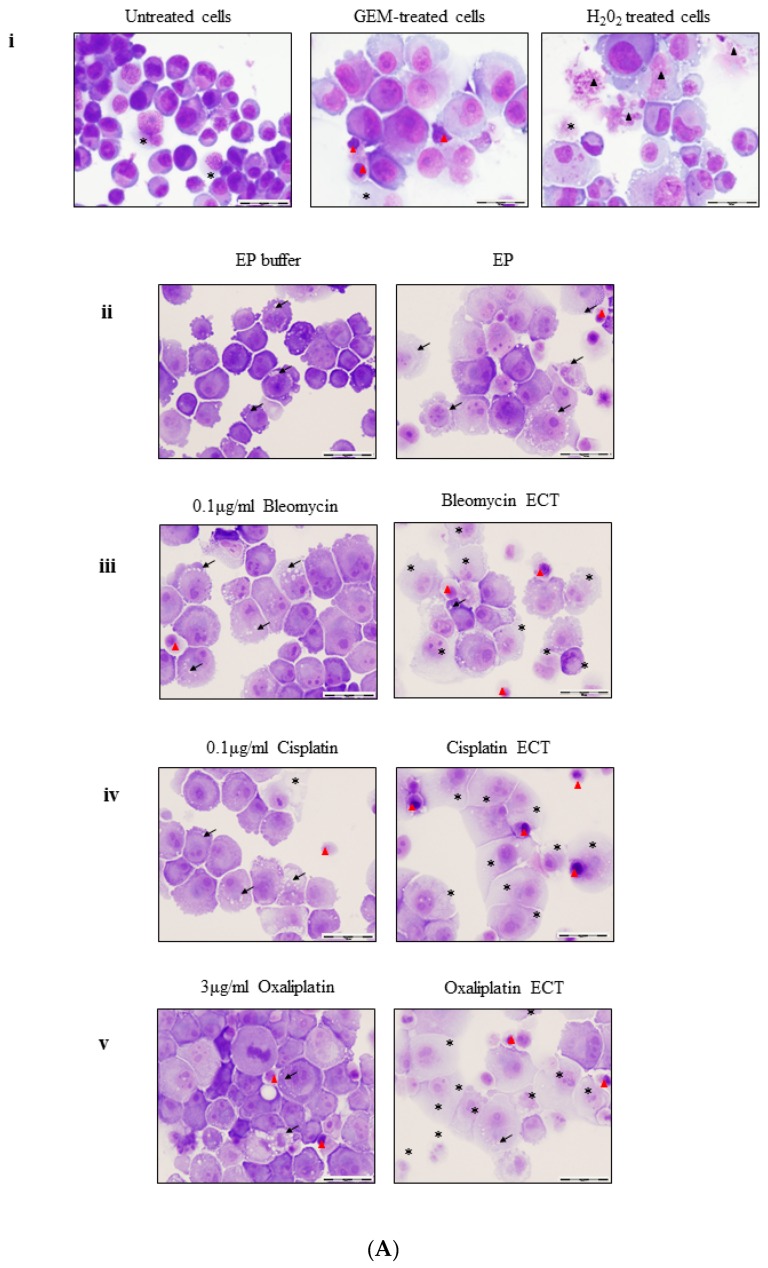

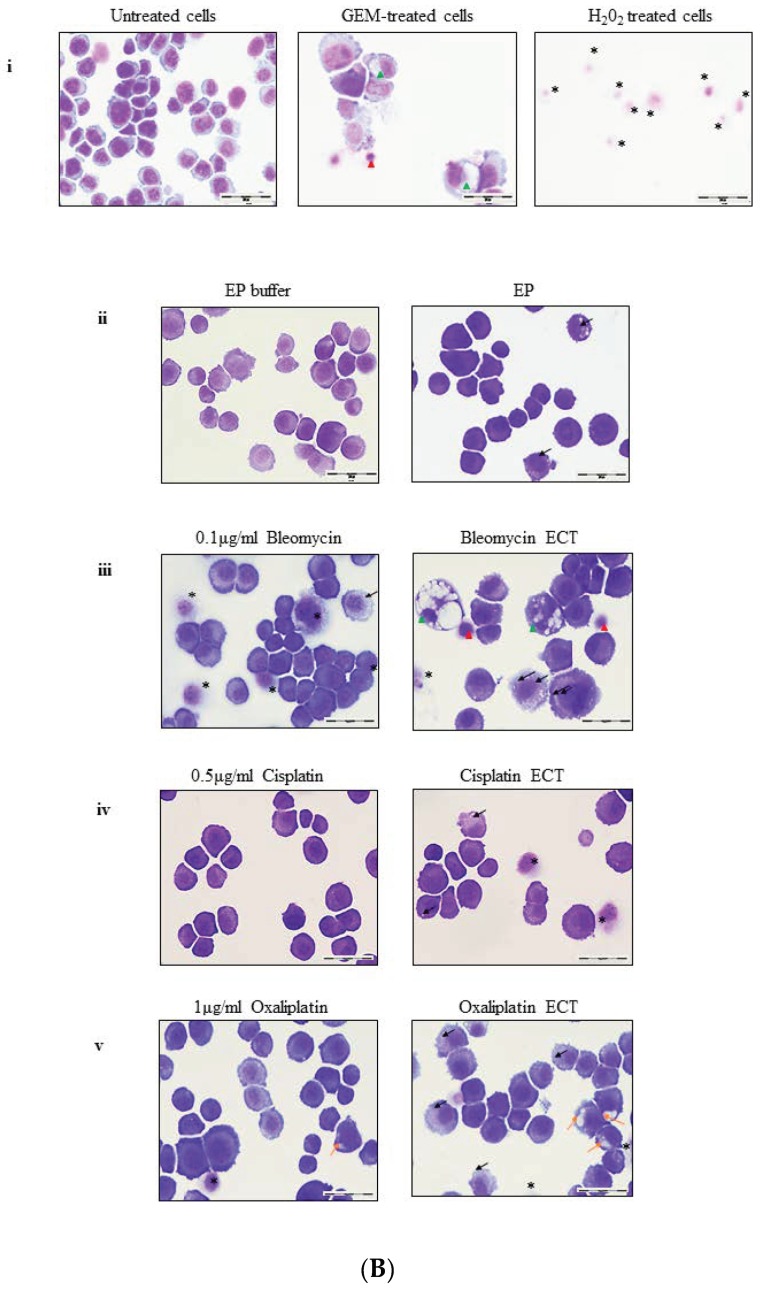
ECT of PANC-1 or Pan02 cells leads to an altered, necrotic-like morphology relative to passive chemotherapy. Morphology of PANC-1 cells (**A**) or Pan02 cells (**B**) either (i) untreated or 72 h post treatment with 100 μM Gemcitabine (GEM), or 0.2% Hydrogen Peroxide (H_2_O_2_), (ii) 24 h following treatment in electroporation buffer alone and electroporated, (iii) in Bleomycin and Bleomycin ECT; (iv) Cisplatin and Cisplatin ECT and (v) Oxaliplatin and Oxaliplatin ECT. PANC-1 cells were treated with 0.1 μg/mL Bleomycin, 0.1 μg/mL Cisplatin or 3 μg/mL Oxaliplatin whereas Pan02 cells were treated with 0.1 μg/mL Bleomycin, 0.5 μg/mL Cisplatin or 1 μg/mL Oxaliplatin. Cells were harvested, cytospun and stained for morphological evaluation using light microscopy. Symbols denote particular cellular characteristics: ▲ = condensed nuclear material, ▲ = decondensed chromatin, ⟶ cytoplasmic vesicles, → perinuclear vacuoles, ▲ = large vacuoles, * = translucent cytoplasm. Scale bar = 20 μm.

Gemcitabine is the standard treatment choice for locally advanced and metastatic pancreatic cancer and it is thought to ultimately cause death by an apoptotic mechanism [21]. We evaluated the morphology of pancreatic cancer cells following ECT treatment, using 100 μM Gemcitabine as a positive control for apoptosis and 0.2% Hydrogen Peroxide (H_2_O_2_), as a control for necroptosis. Consistent with other reports, PANC-1 cells appear to be largely resistant to Gemcitabine [21], with the majority of cells exhibiting normal rounded morphology albeit with a swollen translucent cytoplasm (Figure 2A GEM-treated). Few PANC-1 cells exhibit shrunken morphology, with highly condensed nuclear material (indicated ▲). PANC-1 cells treated with 0.2% H_2_O_2_, known to induce necrosis at high concentrations in epithelial cells [22] contain nuclei with decondensed chromatin (▲) and in some cases, the plasma membrane has completely ruptured (Figure 2A H_2_O_2_ treated).

Incubation of PANC-1 cells in EP buffer leads to an increase in cytoplasmic size with the presence of small cytoplasmic vacuoles in periplasmic locations (indicated by →). Electroporation causes pronounced cell swelling and more cytoplasmic vacuoles (Figure 2Aii). These characteristics are increased upon ECT treatment with 0.1 μg/mL Bleomycin, 0.1 μg/mL Cisplatin and 3 μg/mL Oxaliplatin (Figure 2Aiii-v ECT respectively). Translucent (*) cells are numerous with the occasional small cell with condensed nuclear material (▲).

Unlike PANC-1 cells, the morphology of murine Pan02 cells alters dramatically with Gemcitabine treatment. Cells exhibit clumping of nuclear material and contain large vacuoles (▲) (Figure 2B GEM-treated). Shrunken cells are also apparent (▲). Pan02 cells are barely visible following H_2_O_2_, treatment with all cells highly translucent (*) (Figure 2B H_2_O_2-_treated).

Bleomycin (0.1 μg/mL), Cisplatin (0.5 μg/mL) or Oxaliplatin (1 μg/mL) -treated Pan02 cells do not demonstrate a marked difference in appearance (Figure 2Biii–v respectively). ECT of Pan02 cells with Bleomycin leads them to exhibit morphologies similar to those seen when Gemcitabine-treated (▲ and ▲) (Figure 2Biii ECT). In contrast, ECT with Cisplatin leads to largely unaltered Pan02 cells (Figure 2Biv ECT) whilst Oxaliplatin ECT of Pan02 cells leads to the presence of both the small perinuclear cytoplasmic vesicles as well as larger ones (← and ←) (Figure 2Bv ECT).

Although the signaling pathways and/or biochemical events leading to apoptosis or necrosis are distinct, these cell death modes are accompanied by similar end-stage degradation and disintegration processes, meaning that it is nearly impossible to discriminate between them using a single end-point morphological assessment [23]. ECT causes a reduction in viability (Supplementary Figure S1) and a drop-in recovery at the chosen chemotherapeutic concentrations (Figure 1). However, neither PANC-1 nor Pan02 demonstrate largely classical apoptotic morphological alterations in response to ECT (such the condensation and fragmentation of nuclear material or the presence of apoptotic bodies), suggesting that apoptosis is not the primary mechanism of cell death following ECT in these cell types.

### 2.3. Electroporation-Delivered Bleomycin But not Cisplatin or Oxaliplatin Leads to Effector Caspase Activation in Pancreatic Cancer Cells

The biochemical and morphological changes that are characteristic of apoptosis are considered to be due to the activation of a family of intracellular cysteine–aspartic proteases known as caspases [24]. The activation of the primary executioner, caspase-3 is known to be essential for apoptosis-associated chromatin marginalization, DNA fragmentation, and nuclear collapse. In contrast, necroptosis is tightly regulated by a signaling pathway that is often triggered under conditions of caspase inactivation [25]. To further elucidate the cell death pathway induced in pancreatic cancer cells following ECT, a caspase-3 activity assay was performed.

**Figure 3:**
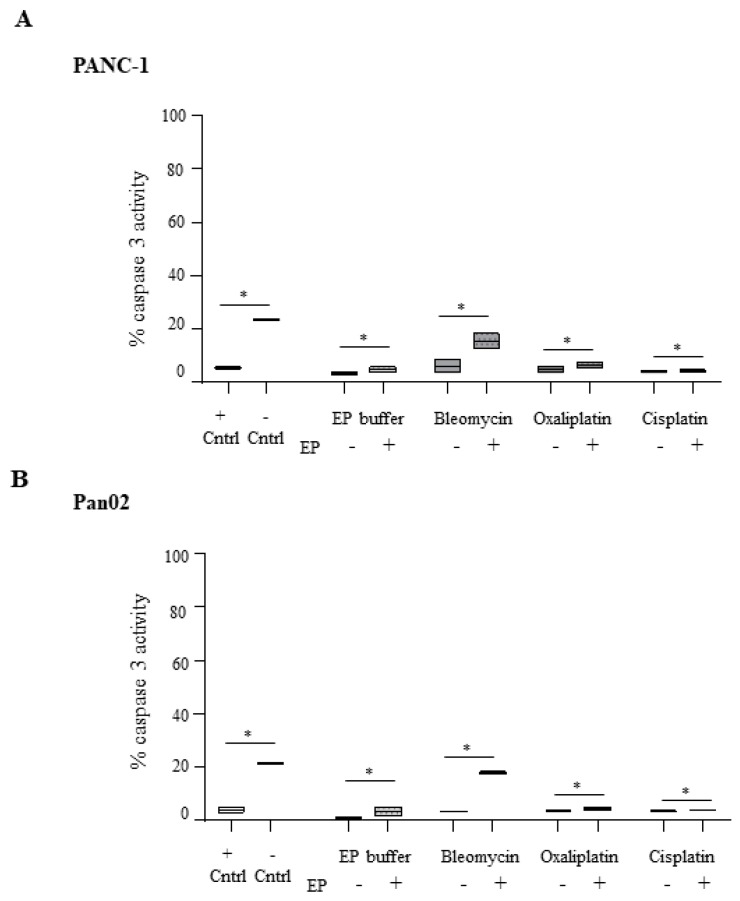
Caspase-3 is activated upon Bleomycin but not platinum drug-based electrochemotherapy in pancreatic cancer cells. PANC-1 (**A**) and Pan02 (**B**) cells were electroporated in the presence or absence of Bleomycin, Cisplatin or Oxaliplatin and subsequently reseeded in 6 wells plates at a density of 0.5 × 10^6^ cells/mL for PANC-1 or 125 × 10^3^ cells/mL for Pan02 in a total volume of 2 mL. For PANC-1 cells, Bleomycin was used at a concentration of 0.1 μg/mL, Cisplatin at 0.1 μg/mL and Oxaliplatin at 3 μg/mL. For Pan02 cells, Bleomycin was used at a concentration of 0.1 μg/mL; Cisplatin at 0.5 μg/mL and Oxaliplatin at 1 μg/mL. 24 h later, intracellular staining of cleaved caspase-3 was performed, and cells analyzed using flow cytometry. OE21 cells were left untreated (negative control) or treated with 30 μM 5-FU for 24 h as a positive control for active caspase-3 activity. The data (minimum to maximum) is also presented as floating bar graphs—with the median integrated intensity relative to EP buffer alone denoted with a line. * statistically significant differences in percentage active caspase activity between the conditions highlighted, * *p* = 0.05.

Treatment of PANC-1 cells with Bleomycin ECT leads to a modest increase in caspase-3 activation which does not reach significance relative to Bleomycin alone (Figure 3A). Treatment with Cisplatin or Oxaliplatin either alone or in combination with EP does not result in caspase-3 activation with activation levels remaining at a basal level (5–7%). Pan02 cells behave very similarly with Cisplatin or Oxaliplatin, both failing to activate caspase-3 irrespective of electroporation (Figure 3B). However, electroporation in the presence of Bleomycin leads to a 4-fold increase in active caspase-3 levels over treatment with the drug alone in both cell lines (*p* = 0.05). Given the dramatic morphological changes seen after Bleomycin ECT treatment of Pan02 cells (Figure 2Biii ECT), it is possible that the catastrophic events associated with this treatment cause dissemination of the internal calcium stores due to organelle damage. Such a rise in free cytosolic calcium levels has been shown to lead to the activation of caspase-3 activity, but this activity was shown to be independent of the apoptosome [26]. Rather, increased intracellular concentrations of calcium have been shown to trigger necroptosis in experimental models [27].

### 2.4. ECT-Induced Cell Death in Pancreatic Cancer Cells Can Be Rescued with Pre-Incubation with a Pan Caspase Inhibitor and Necrostatin-1

Given that the recovery of PANC-1 and Pan02 cells following electroporation in the presence of Bleomycin, Cisplatin and Oxaliplatin is diminished, yet morphologically and biochemically these cells do not appear to undergo classical apoptosis, we sought to investigate alternative cell death mechanisms. Necroptosis is a novel form of programmed cell death that is independent of caspase activity instead requiring the activity of receptor-interacting serine/threonine-protein (RIP) kinases [25]. Necrostatin-1 is an allosteric inhibitor of RIPK1 kinase activity and is widely used to target RIPK1 kinase activity in both human and murine cells [28].

In order to discriminate between apoptotic and necroptotic cell death, cells were stained with Annexin V-FITC and PI and a time course study was performed using flow cytometry. As Gemcitabine failed to elicit distinct apoptotic morphology in PANC-1 cells, an alternative cytotoxic drug, Mitoxantrone (MTX), an inhibitor of DNA topoisomerase II, was used as a positive control for apoptosis induction [29]. Using this assay, cells undergoing apoptosis should move from an Annexin V^−^/PI^−^ state (healthy, viable), to an Annexin V^+^/PI^−^ state, due to the gradual exposure of phosphatidylserine, in a temporal manner. Finally, under in vitro conditions, cells undergoing post apoptotic secondary necrosis eventually end up Annexin V^+^/PI^+^ (double-positive) (Supplementary Figure S2A). Conversely, cells that are truly necroptotic would move directly to a double-positive state [30,31,32] (Supplementary Figure S2B). Four hours following treatment, 35% of MTX-treated PANC-1 cells showed Annexin V^+^/PI^–^ staining compared to untreated cells and this rose to approximately 63% after 18 h without demonstrating concurrent PI-positive staining, suggesting that post-apoptotic secondary necrosis was not induced with MTX within this time frame (Supplementary Figure S3A).

Electroporation alone led to a doubling of the percentage of cells that are Annexin V^+^/PI^+^ over the 18 h time course in PANC-1 cells (Supplementary Figure S3B), from 6 to 12%. Bleomycin alone does not alter the Annexin V^+^/PI^+^ population but Bleomycin-ECT treated PANC-1 cells exhibit a gradual increase over the time period with a tripling of Annexin V^+^/PI^+^ (double-positive) at 18 h. The increase in the Annexin V^+^/PI^+^ (double-positive) population without a concurrent increase in the Annexin V^+^ population indicate that when subjected to ECT, PANC-1 cells move directly to a double-positive state and thus are undergoing de facto necrosis rather than post-apoptotic necrosis.

In order to further investigate the possibility that pancreatic cancer cells die by a regulated cell death mechanism following ECT, we performed inhibitor pre-treatment studies. Pre-treating cells with the pan caspase inhibitor, zVAD.fmk (zVAD) prior to ECT allowed us to further assess the relative contribution of apoptosis (if any) to ECT-mediated cellular demise [33]. Since it has been reported in the literature that treating cells in this manner compels cells to undergo an alternative, caspase-free, method of cell death, Necrostatin-1 (Nec-1) was also added. This small molecule has been reported to allosterically inhibit caspase-independent cell death by preventing receptor-interacting serine/threonine protein kinase 1 (RIPK1) kinase activity [34].

**Figure 4:**
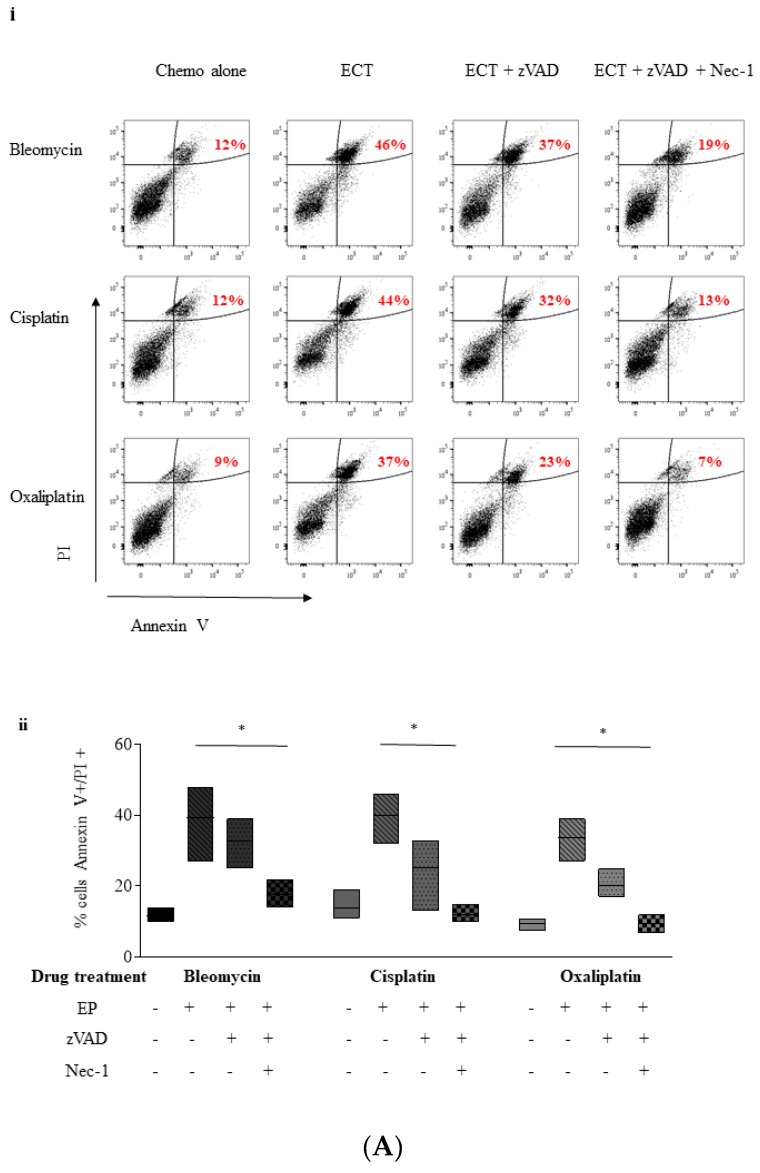

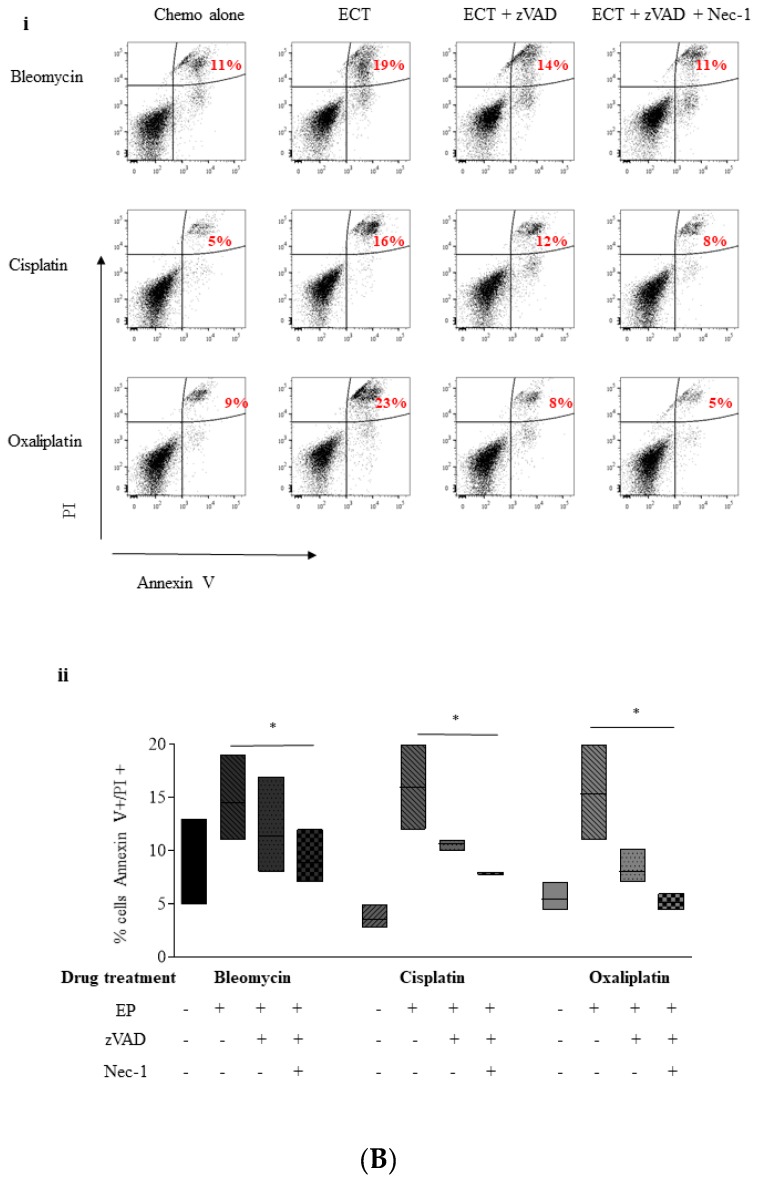
zVAD and Necrostatin-1 pre-treatment reduces the Annexin V positive, PI positive cell population following ECT. (PANC-1 cells (**A**) or Pan02 cells (**B**) were either treated with chemotherapy or electroporated in the presence of chemotherapy, with 25 μM zVAD and/or 10 μM Nec-1. PANC-1 cells were treated with 0.1 μg/mL Bleomycin, 0.1 μg/mL Cisplatin or 3 μg/mL Oxaliplatin whereas Pan02 cells were treated with 0.1 μg/mL Bleomycin, 0.5 μg/mL Cisplatin or 1 μg/mL Oxaliplatin. Cells were subsequently reseeded in 6 wells plates at a density of 0.5 × 10^6^ cells/mL for PANC-1 or 125 × 10^3^ cells/mL for Pan02 in a total volume of 2 mL. After 24 h, all cells (both viable and non-viable) were stained using Annexin V and PI followed by detection using flow cytometry. The experiment was repeated at least three times and the data from one representative experiment is presented as a dot plot (i). The Annexin V positive, PI positive (double-positive) percentage of cells is indicated beside the gated cells in red. The double-positive percentage data from three independent experiments (minimum to maximum) is also presented as floating bar graphs (ii)—with the median integrated intensity relative to EP buffer alone denoted with a line. * statistically significant differences in percentage double-positive cells between the ECT and ECT in the presence of both zVAD and Nec-1 is shown, * *p* = 0.05.

Bleomycin ECT increased the double-positive PANC-1 cell population from 12% to 46% after 24 h relative to drug alone (Figure 4Ai). ECT in the presence of zVAD decreased this population relative to ECT alone by 9% down to 37%. However, the double-positive population in the ECT group was nearly completely rescued by Nec-1 and zVAD (double-positive population returned to 19%) in a statistically significant manner (Figure 4Bii) (*p* = 0.05). Cisplatin and Oxaliplatin ECT of PANC-1 cells also resulted in an increase in the double-positive population which was completely prevented by combining ECT with Nec-1 + zVAD pre-treatment (Figure 4A(i)(ii)) (*p* = 0.05).

The pattern is repeated with Pan02 cells (Figure 4B), with the addition of zVAD and Nec-1 to ECT significantly reducing cell death in all drug treatments compared to ECT alone (Figure 4Bii). Thus, although some cell death as a result of ECT may proceed via a caspase-dependent mechanism, these data implicate an involvement of a second, distinct caspase-independent, RIP1K-mediated mechanism, such as necroptosis.

### 2.5. Inhibition of Necroptosis Combined with ECT Prevents Pancreatic Cell Death and Improves Recovery

#### 2.5.1. Nec-1

**Figure 5:**
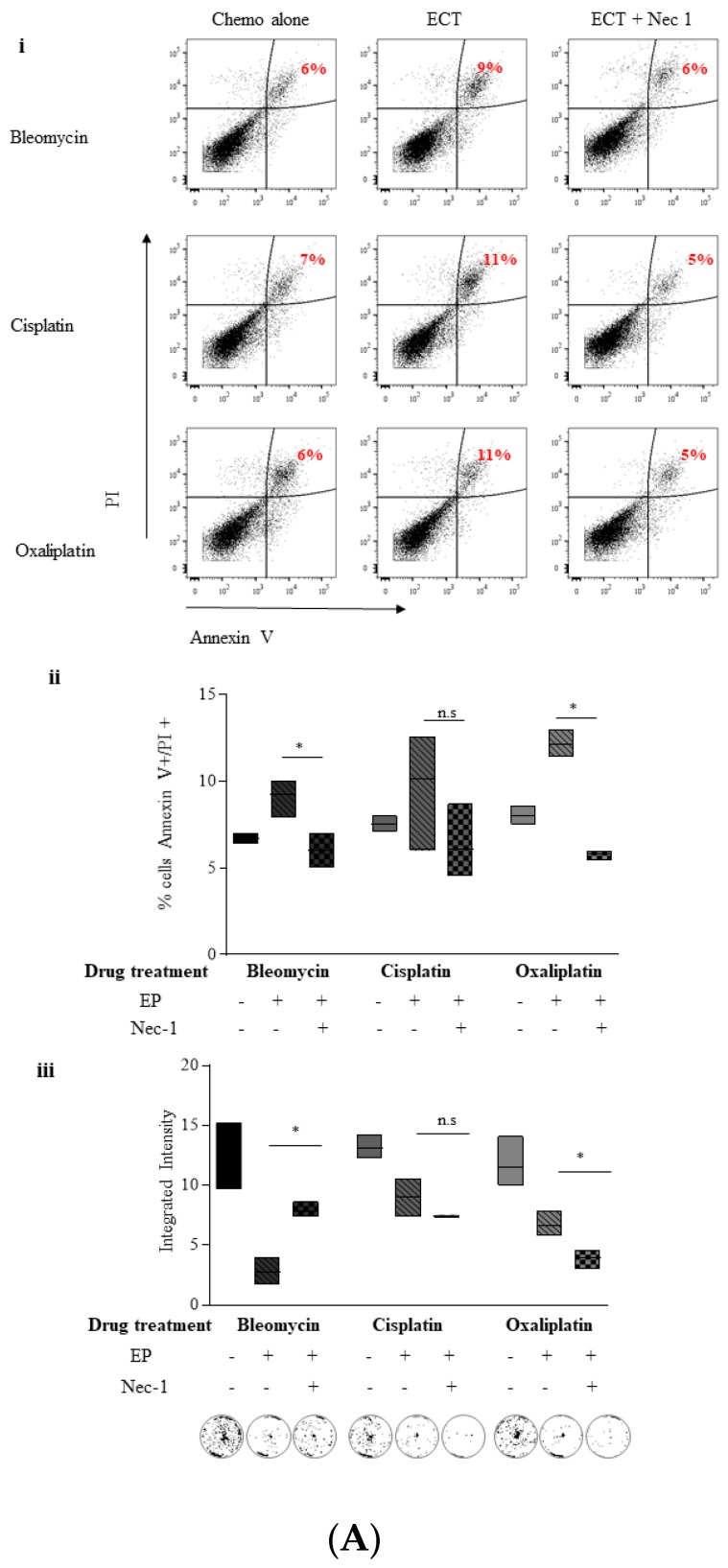

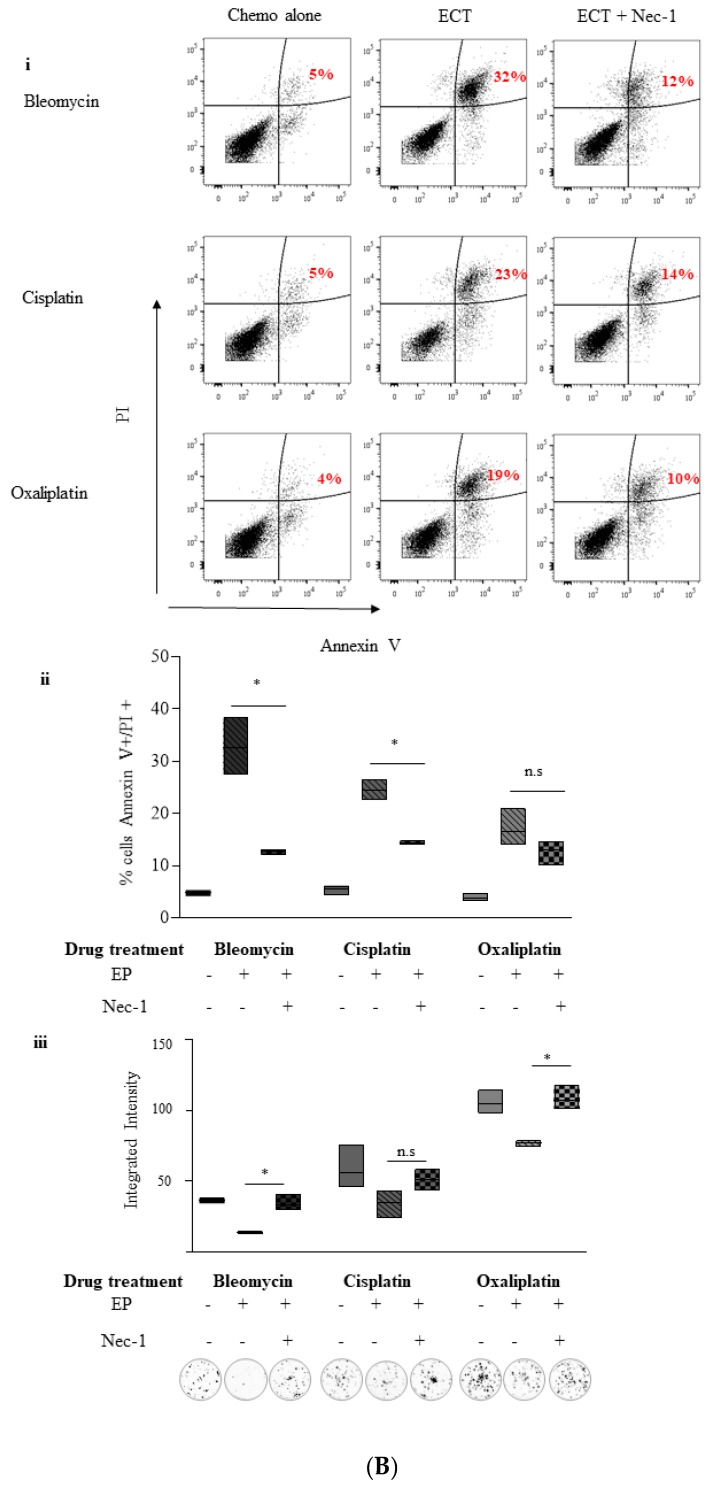
Necrostatin-1 is sufficient to reduce the Annexin V positive, PI positive PANC-1 population following ECT and improves recovery following Bleomycin ECT treatment. (PANC-1 cells (**A**) or Pan02 cells (**B**) were either treated with chemotherapy or electroporated in the presence of chemotherapy, with or without 10 μM Nec-1. PANC-1 cells were treated with 0.1 μg/mL Bleomycin, Cisplatin or 3 μg/mL Oxaliplatin whereas Pan02 cells were treated with 0.1 μg/mL Bleomycin, 0.5 μg/mL Cisplatin or 1 μg/mL Oxaliplatin. Cells were subsequently reseeded in six wells plates at a density of 0.5 × 10^6^ cells/mL for PANC-1 or 125 × 10^3^ cells/mL for Pan02 in a total volume of 2 mL. After 24 h, all cells (both viable and non-viable) were stained using Annexin V and PI followed by detection using flow cytometry. The experiment was repeated at least three times and the data from one representative experiment is presented as a dot plot (i). The Annexin V positive, PI positive (double-positive) percentage of cells is indicated beside the gated cells in red. The double-positive percentage data from three independent experiments (minimum to maximum) is also presented as floating bar graphs (ii)—with the median integrated intensity relative to EP buffer alone denoted with a line. Statistically significant differences in percentage double-positive cells between the ECT and ECT in the presence of Nec-1 is shown, * *p* = 0.05. Cells were counted and 1 million cells were resuspended in EP buffer and electroporated either in the presence or absence of Nec-1 and chemotherapy. 24 h later, 1000 cells (PANC-1) or 400 cells (Pan02) were seeded in triplicate in 6 well plates in complete media. The recovery of cells post treatment was quantified by fluorescent intensities of colonies formed 14 days later. Each well is a representative of at least 9 similar wells (3 independent experiments). The data from three independent experiments (minimum to maximum) is also presented as floating bar graphs (iii) with the median integrated intensity relative to EP buffer alone denoted with a line. * statistically different differences in the number of colonies formed between ECT and ECT with Nec-1 pre-treatment when cells were allowed to recover, *p = 0.05, n.s = non-significant.

In order to further investigate necroptosis as a mode of cell demise following ECT, and to confirm that this type of cell death was not simply a consequence of caspase inhibition, we conducted Annexin V/PI staining on PANC-1 cells that had been pre-treated with Necrostatin-1 (Nec-1) only. Pre-treatment with Nec-1 alone under Bleomycin ECT conditions reduces the Annexin V^+^/PI^+^ (double-positive) population from 9% to 6% (Figure 5Ai Bleomycin).

A similar pattern is seen with the platinum drugs, (Figure 5Ai, Cisplatin, Oxaliplatin) and the decrease is significant (*p* = 0.05) with all three drugs (Figure 5Bii) suggesting a *bona fide* role for the necroptotic pathway in the PANC-1 response to ECT treatment. Morphologically, Nec-1 pre-treatment appears to have a protective effect on cells electroporated in the presence of all three drugs with the majority of cells largely regaining a more normal appearance 24 h following treatment (Supplementary Figure S4A).

We then examined the ability of pancreatic cancer cells to recover post ECT treatment when pre-treated with Nec-1. PANC-1 cells showed improved recovery post Bleomycin ECT (*p* = 0.05) (Figure 5Aiii). However, PANC-1 cells pre-treated with Nec-1 during electroporation and also during recovery did not recover following ECT with Cisplatin and Oxaliplatin (Figure 5Aiii Cisplatin, Oxaliplatin). These data suggest that although Nec-1 appears to offer some short-term protection against the cytotoxicity of the drugs delivered by electroporation. The DNA-damaging effects of the drugs used on PANC-1 cells are not prevented by this inhibitor. It may also indicate a particular sensitivity of this cell line to Nec-1—indeed there are reports that Nec-1 itself is capable of inhibiting cell proliferation, being toxic at low doses [35].

The experiments were repeated with Pan02 cells—Nec-1 alone was able to rescue Pan02 cells from Bleomycin, Cisplatin, and Oxaliplatin ECT-induced Annexin V^+^/PI^+^ staining, reaching significance in the case of Bleomycin (*p* = 0.05) and Cisplatin (*p* = 0.05) (Figure 5Bi,ii). Morphologically, pre-treatment with Nec-1 appears to protect cells from ECT-induced changes, with the majority of Pan02 cells retaining their normal, untreated appearance (Supplementary Figure S4B). Finally, Nec-1 was able to protect Pan02 cells from ECT-induced reductions in survival following treatment with any of the three drugs (*p* = 0.05 Bleomycin, Oxaliplatin) (Figure 5Biii).

#### 2.5.2. Necrosulphonamide (NSA)

In order to confirm a potential role for necroptosis in the response of PANC-1 cells to ECT, cells were pre-treated with NSA, a highly potent, human-specific, selective inhibitor of MLKL, a phosphorylation substrate of RIPK3 [36,37]. NSA acts specifically to prevent the RIP1/RIP3/MLKL necrosome formation and, unlike Nec-1, has been demonstrated to have no other off-target effects which may have influenced PANC-1 cell survival in Figure 5Aiii [37]. NSA pre-treatment was able to prevent a reduction in PANC-1 cell viability post ECT in a statistically significant manner for Bleomycin, Cisplatin, and Oxaliplatin (*p* = 0.01) (Supplementary Figure S5A). Furthermore, NSA was able to protect PANC-1 cells from any ECT-mediated reductions in survival (Supplementary Figure S5B).

## 3. Discussion

With few patients suitable for curative surgery, and most having only a limited response to chemotherapy, pancreatic cancer (PC) patients face a bleak prognosis [2]. The pancreas is surrounded by vital structures and thermal ablation techniques which could be potentially useful in debulking the tumour have the potential to cause inadvertent damage to the duodenum or peripancreatic vessels [38]. This has led to a search for alternative non-thermal ablative therapy for use in pancreatic cancer. In the last few years, methods in which short, high-voltage pulses are applied to tissues to permeabilize the cell membranes have been tested in clinical trials in order to selectively deliver chemotherapy to tumours. The technique, known as electrochemotherapy (ECT), describes temporary and reversible permeabilization of the plasma membrane in the presence of a suitable chemotherapy [39]. Following the inaugural ESOPE (European Standard Operating Procedures on Electrochemotherapy) study, a phase II trial conducted in 2006 in which the clinical application of ECT was standardized for use in small cutaneous tumours, ECT became a routine clinical treatment for melanoma [14]. The revised ESOPE, published in 2018, reports of the success physicians have had using ECT to treat a number of solid tumours including adenocarcinomas, basal cell carcinomas, squamous cell carcinoma, and sarcomas [40]. Development of endoscopic devices such as the Endove or expandable probes [41] would give ECT the potential to effectively treat tumours that are surrounded by vital structures such as larger blood vessels, nerves, and viscera without damage to them and without side effects or major complications, so rendering PC a viable candidate [42]. Indeed, ECT has already been used in a number of preliminary studies in PC with promising results [43].

Well characterized for its role as an apoptotic inducer, early in vitro studies suggested that, under certain conditions, TNFα could also mediate death that demonstrated the morphological features of necrosis. This was surprising, given that necrosis was until that point considered an accidental form of cell death [44]. Further studies led to the understanding that the kinase activity of RIPK1 and inhibition of caspase-8 provided the precise conditions for this TNF α-mediated form of regulated necrotic cell death [45]. Since then, numerous investigations have begun in earnest to determine the precise molecular events leading to this type of cellular demise, termed ‘necroptosis’ in 2005, following the identification of ‘Nec-1′ a small molecular inhibitor of TNF α-mediated necrosis [34]. It has since been demonstrated that necroptosis is part of the arsenal of physiological responses *in vivo*, resulting from multiple triggers such as activation of other death receptors (namely FasL and TRAIL), interferons, Toll-like receptors and virally activated pathways, rather than simply an artefact of caspase activation in vitro [46,47,48]. However, to date, mechanistically, TNF α- induced necroptosis is still the best understood. The core event in necroptosis is the formation of the detergent-insoluble ‘necrosome’ complex of homologous Ser/Thr kinases, receptor protein interacting kinase 1 (RIPK1) and receptor interacting protein kinase 3 (RIPK3) [49]. The necrosome promotes phosphorylation of a key pro-death effector molecule known as mixed lineage kinase domain-like (MLKL), by RIPK3. MLKL is the critical effector molecule in this necroptotic pathway, the conformational changes associated with its activation leading to the disruption of the plasma membrane that is typical of necrosis [36].

Many cancers show resistance to apoptosis and induction of RIPK-dependent necroptosis may provide an alternative avenue to eliminate cancer cells. For example, one could circumvent the resistance of cancer cells to apoptosis by simultaneously inhibiting the ‘brakes’ on apoptosis such as those imposed by cIAP1/2 and preventing caspase activation. For example, BV6 has been shown to be effective in killing apoptosis resistant pancreatic cancer cells when combined with Zvad.fmk [50].

The promotion of necroptosis could act to enhance immune surveillance and therefore encourage an immune response to cancer. Indeed, it has been shown that necroptotic cells produce signals cross priming CD8+ T cells by dendritic cells in a manner that requires both DAMP release and RIP1K NFκ B dependent proinflammatory transcription in the dying cells [51,52]. Encouragingly, necroptotic murine colon carcinoma cells have been used to vaccinate against tumours [53].

Alternatively, inhibition of necroptosis could reduce cancer-induced inflammation. Recent data suggest that PDAC cells have high expression levels of RIPK1 and RIPK3 and are therefore potentially particularly sensitive to necroptosis [13]. However, somewhat counterintuitively, the expression of RIPK1 and RIPK3 was tumour promoting in this case. Expression levels of the chemokine, CXCL-1 was high in the model and patient samples and was further enhanced by Gemcitabine treatment indicating a key role of necrosomes in driving CXCL1 expression. Furthermore, loss of RIPK3 or chemical inhibition of necroptosis significantly reduced tumorigenesis in their tumour model. Mechanistically, this was linked to an increased RIPK1/RIPK3-dependent protein product of PDA cells (SAP130), which promoted cellular immune suppression by tumour infiltrating macrophages expressing the protein receptor (Mincle) that were themselves recruited in a CXCL1-dependent manner. This study provided a clear illustration of the tumour promoting activity of RIPK1/RIPK3-mediated inflammation via myeloid-dependent immune suppression [13]. Gemcitabine/nab paclitaxel and FOLFIRINOX combination chemotherapy regimens are the two standards of care first-line treatment regimens for advanced pancreatic cancer in the clinic presently [54]. It is therefore interesting to note that Gemcitabine, has been shown to increase RIPK1 and RIPK3 expression [13], thereby, potentially promoting tumour progression in PDAC patients, albeit unintentionally. This may go some way to explain the apparent resistance demonstrated in the majority of patients undergoing Gemcitabine treatment, especially historically as a single agent.

The complexity of the role of RIPK1 and RIPK3 in PDAC is highlighted further by the fact that although chemical inhibition of RIPK1 by Nec-1 led to reduced necroptosis in PDAC cells, knocking down the entire protein did not have the same effect [13]. It is thought that signals that fundamentally require RIPK1 signaling are rewired in its absence thus bypassing it entirely and directly engaging the downstream effector molecule, RIPK3 instead. These data suggest that the necroptosis-relevant genes and proteins may have differing contributions to tumorigenesis (targeted for cancer prevention) and later tumour progression in PDAC (which would be targeted for treatment). Accordingly, caution needs to be applied considering their manipulation in a cancer setting.

## 4. Materials and Methods

### 4.1. Cell Culture

The established human pancreatic cancer cell line PANC-1 cells were obtained from the American Type Culture Collection (ATCC). PANC-1 cells were chosen as they exemplify exocrine pancreatic ductal adenocarcinoma (PDAC). PDAC is one of the most aggressive and lethal forms of the disease and they comprise over 90% of all pancreatic cancers diagnosed. PANC-1 cells have been used as a model for functional and pathway studies since their initial culture over 40 years ago [55]. Pan02 cells, obtained from the National Cancer Institute at Frederick (Massachusetts, USA), were included in this study due to their high metastatic potential [56]. PANC-1 cells were maintained in Dulbeccos Modified Media supplemented with 10% (*v*/*v*) fetal calf serum, whilst Pan02 cells were maintained in RPMI 1640 medium with 10% (*v*/*v*) fetal calf serum. The human oesophageal cancer cell line OE21 was obtained directly from the European Collection of Cell Cultures and was maintained in RPMI 1640 medium with 10% (*v*/*v*) fetal calf serum. All cell lines were supplemented with 1% penicillin/streptomycin, and were grown at 37 °C, 5% CO_2_.

### 4.2. PI Exclusion Assay to Measure Viability

Propidium iodide (PI) is a small fluorescent molecule that binds to DNA but is unable to passively traverse into cells that possess an intact plasma membrane. PI uptake versus exclusion can thus be used to discriminate dead cells, in which plasma membranes become permeable, from live cells with intact membranes. Cells were seeded at 0.5 × 10^6^ (PANC-1) and 0.25 × 10^6^ (Pan02) cells per 6-well plate, for 24 h post-treatment respectively with Cisplatin (Teva Pharma., Petah Tikva, Israel.), Oxaliplatin (Accord Healthcare Ltd., Cork, Ireland) or Bleomycin (Nippon Kayaku Co Ltd., Tokyo, Japan) either alone or in combination with electroporation. Cells were typsinised, washed in PBS and then PI was added at 5 μg/100 uL. Samples were then analyzed on the BD LSR II flow cytometer.

### 4.3. Electroporation

Following harvesting by trypsinisation, 1 million cells were resuspended in 400 µL of HEPES electroporation buffer (10 mM HEPES (Lonza, Basel, Switzerland), 250 mM sucrose and 1 mM MgCl_2_ in sterile water). Cells were electroporated in 4 mm cuvettes (VWR) in the presence of Bleomycin (0.1 μg/mL for both PANC-1 and Pan02), Cisplatin (0.1 μg/mL for PANC-1 and 0.5 μg/mL for Pan02) or Oxaliplatin (0.3 μg/mL for Panc-1 and 0.2 μg/mL for Pan02). Cells were incubated at 37 degrees Celsius for 30 min before dilution in a further 600 μL of EP buffer. The electroporation parameters were as follows: 8 pulses of 99 μs at a frequency of 1 Hz with 0.6 kV/cm for PANC-1 cells or 1 kV/cm for Pan02 cells using a BTX electroporator. Parameters for each cell line were optimized for high permeabilization and low cell death from electroporation alone.

### 4.4. Colony Formation Assay

The colony formation assay measures the proliferative capacity of treated adherent single cells to form distinct colonies on a monolayer surface, thus indirectly measuring recovery following treatment regimes.

We assessed the ability of cells to recover the following electroporation with Bleomycin, Cisplatin, Oxaliplatin or electroporation alone and to form colonies. 24 h post-treatment, either 250 cells/mL PANC-1 or 100 cells/mL Pan02 cells, in a total of 4 mL were reseeded into a well of a six-well plate (in triplicate). Cells were allowed to adhere and grow for between 10 and 14 days. To visualize colonies, media was removed, cells were fixed in 96% ethanol for 10 min and stained with Prodiff solution C (Braidwood Laboratories, London, UK). Plates were scanned using the Odyssey IR imaging system (Li-Cor, Cambridge, United Kingdom) to quantify the number of colonies.

### 4.5. Evaluation of Caspase-3 Activity

24 h following treatment, cells were fixed in 2% para-formaldehyde (PFA), and then washed in a permeabilization buffer (0.1% Triton X-100, 0.1% sodium azide, 10 mM HEPES, 4% FCS, 150 mM NaCl). Cells were then incubated with a primary rabbit polyclonal anti-active caspase-3 antibody reactive against both human and murine samples (559565 BD Biosciences, NJ, USA) for 1 h at room temperature. A secondary only control was used to mark the level above which fluorescence intensity was considered to be specific. Samples were then incubated with an anti-rabbit FITC conjugated secondary antibody (Life Technologies, CA, USA), washed and subsequently run on the BD LSR II flow cytometer and data generated was analyzed by FACSDIVA software. Apoptosis competent OE21 cells, either left untreated or incubated with 30 μM 5-FU for 24 h were run with each experiment to verify known active caspase-3 activity.

### 4.6. Evaluation of Morphology

Morphological features of cells treated with Bleomycin, Cisplatin, and Oxaliplatin were examined by light microscopy. Aliquots of non-electroporated non drug treated (untreated control cell population), drug-treated and electrochemotherapy-treated (ECT) cells were cytospun onto glass slides and stained using Pro-Diff (Braidwood Laboratories, London, UK). Briefly, cells were fixed and stained with buffered eosin followed by methyl thionins. Cells with a condensed nucleus and demonstrating blebbing of an intact plasma membrane were considered to be apoptotic whilst those cells that displayed an increase in cell volume; an unchanged nuclei size but with a disrupted plasma membrane were counted as necrotic cells. Cells treated with 100 μM Gemcitabine (GEM) and 0.2% Hydrogen Peroxide (H_2_O_2_) were used as controls for apoptosis and necrosis respectively. Cytospin images are representative of at least three independent experiments.

### 4.7. Analysis of Phosphatidylserine (PS) Exposure Versus Cell Permeability by Flow Cytometry

Annexin V, a member of the phospholipid binding Annexin family, binds to phosphatidylserine that normally resides in the inner plasma membrane of viable cells but is rapidly externalized when the cell is exposed to pro-apoptotic stimuli. Combining Annexin V with PI staining can help to aid in the discrimination between apoptotic and necroptotic cell populations. This discrimination relies on the fact that apoptotic cells will first become Annexin V positive (remaining PI negative) followed by subsequent PI uptake when the nuclear material finally becomes exposed. In contrast necrotic cells by virtue of their rapid lytic nature, go directly to an Annexin V/PI double-positive state [16,17,23]. To discriminate between apoptosis and necrosis, following treatment with electroporation buffer alone, electroporation alone, drug alone or ECT, cells were seeded. 24 h later cells were typsinised, washed and then resuspended in 95 μL of binding buffer and incubated with 5 μg/mL Annexin V-FITC (ThermoFisher, MA, USA) for 20 min at room temperature, protected from light. Cells were then washed in binding buffer, resuspended in the same and PI added at a final concentration of 2.5 μg/mL. Cells were run immediately on the BD LSR II flow cytometer and data generated analyzed using FACS Diva software. The analysis was performed by generating a dot plot of Annexin V (FL1) versus PI (FL3) and defining quadrants that divide cell populations according to Annexin V and PI positivity.

### 4.8. Inhibitor Pre-Treatment

In order to determine the relative proportion of cell death that can be attributed to caspase-dependent apoptosis, prior to ECT, cells were pretreated with carbobenzoxy-valyl-alanyl-aspartyl-[O-methyl]- fluoromethylketone (zVAD.fmk), a cell-permeant pan caspase inhibitor that irreversibly binds to the catalytic site of caspase proteases and can inhibit the induction of apoptosis [33]. By combining this inhibitor with Necrostatin-1 (Nec-1, methylthiohydantoin-tryptophan)—a selective type III allosteric inhibitor of death domain receptor-associated adaptor kinase RIP1 [34], we were able to determine the proportion of cell death that can be attributed to necroptosis. In order to discriminate between genuine necroptosis as opposed to that which is contingent on caspase inactivation, cells were pre-treated with Nec-1 alone. Cells were incubated with 25 μM zVAD-fmk and/or 10 μM of Nec-1 for 30 min prior to electroporation. Necrosulfanamide (NSA) specifically blocks necroptosis downstream of RIP3 activation by preventing the MLKL-RIP3 interaction in human cells [37]. PANC-1 cells were pre-treated with 1 µM NSA for 1 h prior to electroporation.

### 4.9. Statistical Analysis

Data from three independent experiments are presented as floating bars using minimum and maximum values with a line at the median. Asterisks indicate the level of significance—* p = 0.05, n.s. non-significant.

## 5. Conclusions

In the data presented here, we have demonstrated that pancreatic cancer cells are sensitised to the cytotoxic effects of Bleomycin, Oxaliplatin and Cisplatin when they are delivered by electroporation. Specifically, ECT negatively affects the ability of pancreatic cancer cells lines to recover from treatment with these chemotherapies and necroptosis may well be the preferred mechanism for PDAC cell death following ECT. Necroptotic inhibition, but not apoptotic inhibition, was sufficient to prevent ECT-mediated cell death. This obviously has important consequences when contemplating new therapeutic opportunities for patients with this devastating disease. Focusing efforts on inhibition of RIPK1 kinase activity in PDAC seems to be justified, given the tolerability of newer necroptotic inhibitors such as Nec-1s [57], the lack of a remarkable phenotype in kinase-dead animals [58], and that effective protection has been observed in numerous animal models of human pathology including PDAC upon inhibition of RIPK1 kinase activity.

## Supplementary Materials

The following are available online at https://www.mdpi.com/2072-6694/11/8/1177/s1, Figure S1: The viability of pancreatic cancer cells is decreased 24 h following ECT treatment. Figure S2: Analysis of PS exposure versus cell permeability using Annexin V and PI staining by flow cytometry. Figure S3 PANC-1 cells undergoing cell death following MTX treatment move to an Annexin V positive stage whilst cells following Bleomycin ECT move directly to a double-positive (Annexin V+/PI+) state. Figure S4 Necrostatin-1 is sufficient to rescue cell morphology from ECT-mediated alterations. Figure S5 NSA inhibits ECT-mediated death in PANC-1 cells and improves their recovery.
