# Supplementary material for: Electrochemotherapy Causes Caspase-Independent Necrotic-Like Death in Pancreatic Cancer Cells
Source: Cancers (Basel). 2019 Aug 14;11(8):1177. doi: 10.3390/cancers11081177 (PMC6721532; doi:10.3390/cancers11081177)
Supplement: Supplementary file 1 [file cancers-11-01177-s001.zip › Supplementary Figure Legends.docx]

Supplementary Figure 1

**The viability of pancreatic cancer cells is decreased 24 hours following ECT treatment.**

PANC-1 **(A)** and Pan02 **(B)** cells were treated with chemotherapy alone or in combination with electroporation (+EP). PANC-1 cells were either treated with 0.1μg/ml Bleomycin or Cisplatin and 3μg/ml Oxaliplatin. Pan02 cells were either treated with Bleomycin at 0.1μg/ml; Cisplatin at 0.5μg/ml and Oxaliplatin at 1μg/ml. After 24 hours, viability was assessed by PI uptake using flow cytometry. Data from three independent experiments are presented as bar graphs. *statistically significant differences in viability, **p* ≤ 0.05, ***p* ≤0.01.

Supplementary Figure 2

**Analysis of PS exposure versus cell permeability using Annexin V and PI staining by flow cytometry.**

Analysis of Annexin V/PI stained cells by flow cytometry allows for quantitation of cells that are Annexin V negative, PI negative (live cells); Annexin V positive, PI negative (early apoptotic); Annexin V positive and PI positive (late apoptotic and necrotic). During apoptosis (**A**), there is a time lag that occurs between cells becoming positive for PS exposure and the loss of membrane integrity. This means that the rapid membrane changes that leading to PS exposure lead to the majority of the cells moving from the lower left quadrant (PI negative, Annexin V negative) to the lower right quadrant (PI negative, Annexin V positive). After some time, the membrane integrity becomes compromised and cells become positive for PI and the cell population then shifts to the upper right quadrant (PI positive, Annexin V positive). By contrast, during necrosis (or necroptosis) (**B**), the exposure of PS coincides with outer membrane permeabilization. Thus cells move directly from the lower left quadrant (PI negative, Annexin V negative) to the upper right quadrant (PI positive, Annexin V positive) without passing through an intermediate (PI negative, Annexin V positive) stage. A cartoon representation of the cell population and it’s movement over time is shown above. Figure adapted from [1]

Supplementary Figure 3

**PANC-1 cells undergoing cell death following MTX treatment move to an Annexin V positive stage whilst cells following Bleomycin ECT move directly to a double positive (Annexin V+/PI+) state.**

PANC-1 cells were either **(A)** untreated or treated with 1μM Mitoxantrone (MTX) as a control for apoptosis induction or **(B)** electroporated in the presence or absence of 0.1μg/ml Bleomycin. Samples of cells were taken after 4, 6 and 18 hours respectively, and were stained using Annexin V and PI followed by detection using flow cytometry. The percentage of Annexin V positive, PI negative cells is indicated in the lower right quadrant for MTX treated cells in red (**A**) whilst the Annexin V positive, PI positive (double positive) Bleomycin or Bleomycin ECT percentage of cells is indicated beside the gated cells in red (**B**).

Supplementary Figure 4

**Necrostatin-1 is sufficient to rescue the morphology of PANC-1 cells from ECT-mediated alterations.**

Morphologies of PANC-1 cells **(A)** or Pan02 cells **(B)** 24 hours following treatment in either **(i)** in EP buffer alone, or electroporated; **(ii)** in Bleomycin, Bleomycin ECT, Bleomycin ECT in the presence of Nec-1; **(iii)** Cisplatin and Cisplatin ECT, Cisplatin ECT in the presence of Nec-1 and **(iv)** Oxaliplatin and Oxaliplatin ECT, Oxaliplatin ECT in the presence of Nec-1. PANC-1 cells were treated with 0.1μg/ml Bleomycin, Cisplatin or 3μg/ml Oxaliplatin whereas Pan02 cells were treated with 0.1μg/ml Bleomycin, 0.5μg/ml Cisplatin or 1μg/ml Oxaliplatin. Nec-1 was used at a concentration of 10μM. Cells were harvested, cytospun and stained for morphological evaluation under light microscopy. Symbols denote particular cellular characteristics: = condensed nuclear material, = decondensed chromatin, = large vacuoles, 🡪 cytoplasmic vesicles, 🡪 perinuclear vacuoles, * = translucent cytoplasm. Scale bar = 20μm.

Supplementary Figure 5

**NSA is inhibits ECT-mediated death in PANC-1 cells and improves their recovery.**

PANC-1 were either treated with chemotherapy alone, in combination with electroporation (+ EP) or electroporated following pre-treatment with 1μM Necrosulphanamide (+NSA +EP). After 24 hours, viability was assessed by PI uptake using flow cytometry. Data from three independent experiments are presented as bar graphs **(A)**. *statistically significant differences in viability, ***p* ≤0.01, n.s non significant.

Cells were counted and 1 million cells were resuspended in EP buffer and electroporated either in the presence or absence of NSA and chemotherapy. Cells were either treated with 0.1μg/ml Bleomycin or Cisplatin and 3μg/ml Oxaliplatin. NSA was used at a concentration of 1μM. 24 hours later, 1000 cells were seeded in triplicate in complete media. The recovery of PANC-1 cells post treatment was quantified by fluorescent intensities of colonies formed 14 days later. Each well is a representative of at least 6 similar wells (2 independent experiments).
