# Supplementary figures and images for: Electrochemotherapy Causes Caspase-Independent Necrotic-Like Death in Pancreatic Cancer Cells
Source: Cancers (Basel). 2019 Aug 14;11(8):1177. doi: 10.3390/cancers11081177 (PMC6721532; doi:10.3390/cancers11081177)

Supplementary Figure 1

A PANC-1

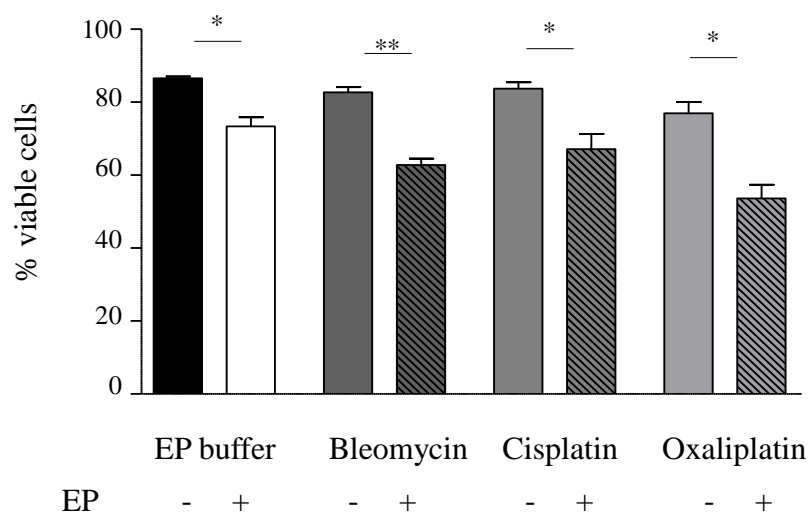

B Pan02

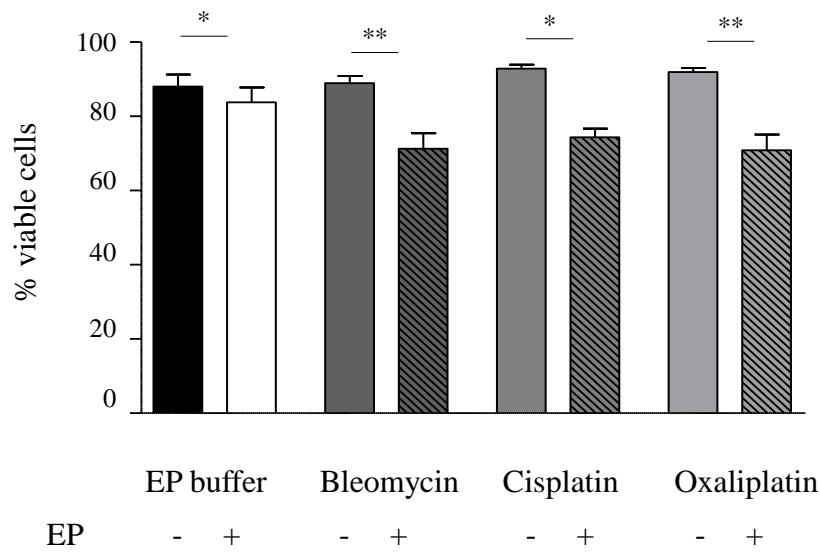

Supplement: Supplementary file 1 [file cancers-11-01177-s001.zip › SD 1.pdf]

Supplementary Figure 2

A

Apoptosis

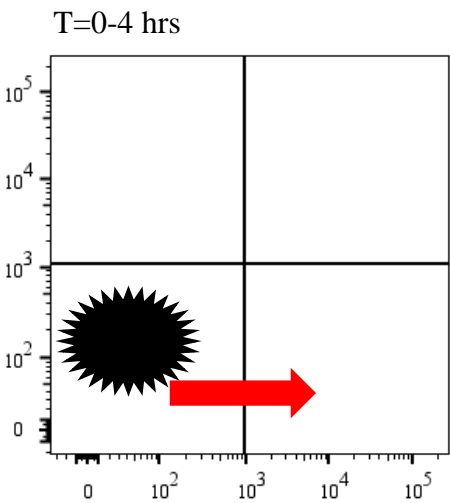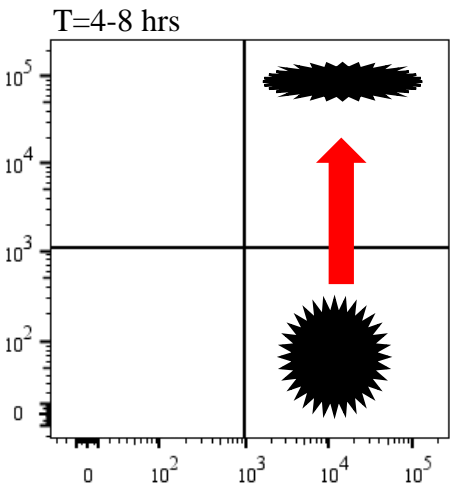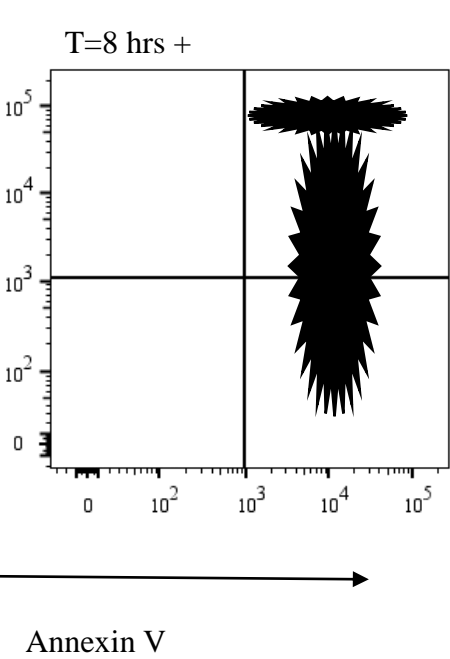

B

Necrosis

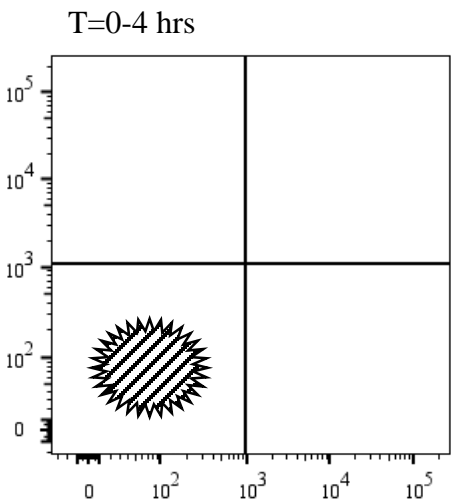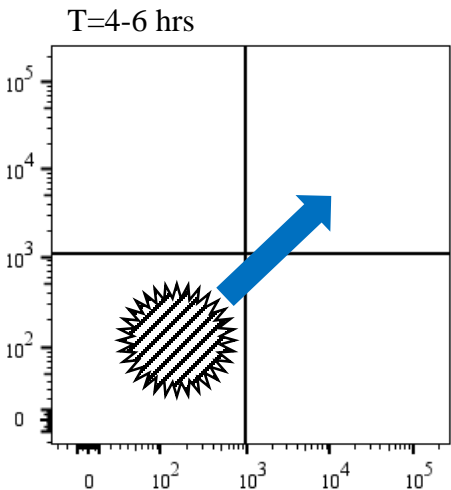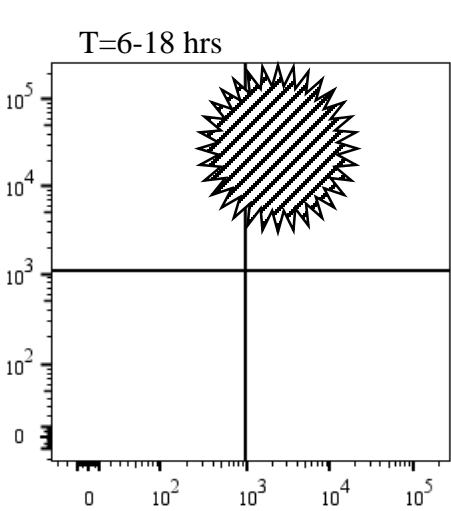

Supplement: Supplementary file 1 [file cancers-11-01177-s001.zip › SD 2.pdf]

Supplementary Figure 3

A

4 hrs

6 hrs

18 hrs

PI

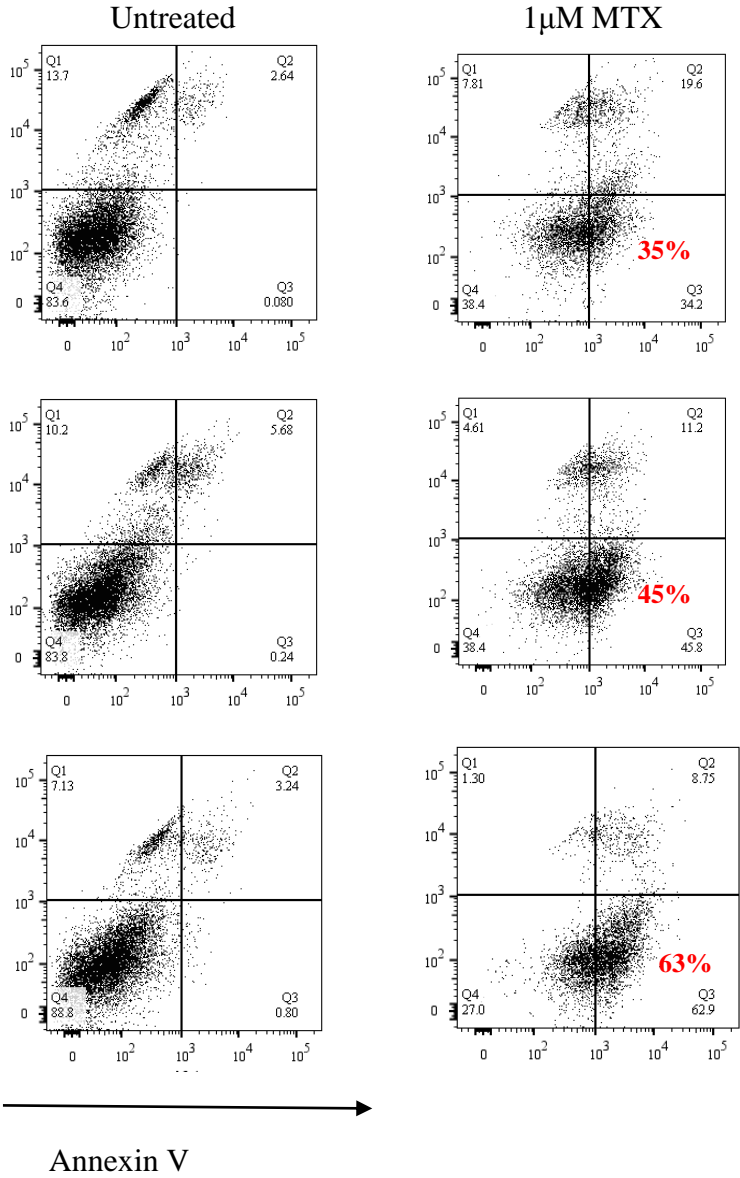

B

4 hrs

6 hrs

18 hrs

PI

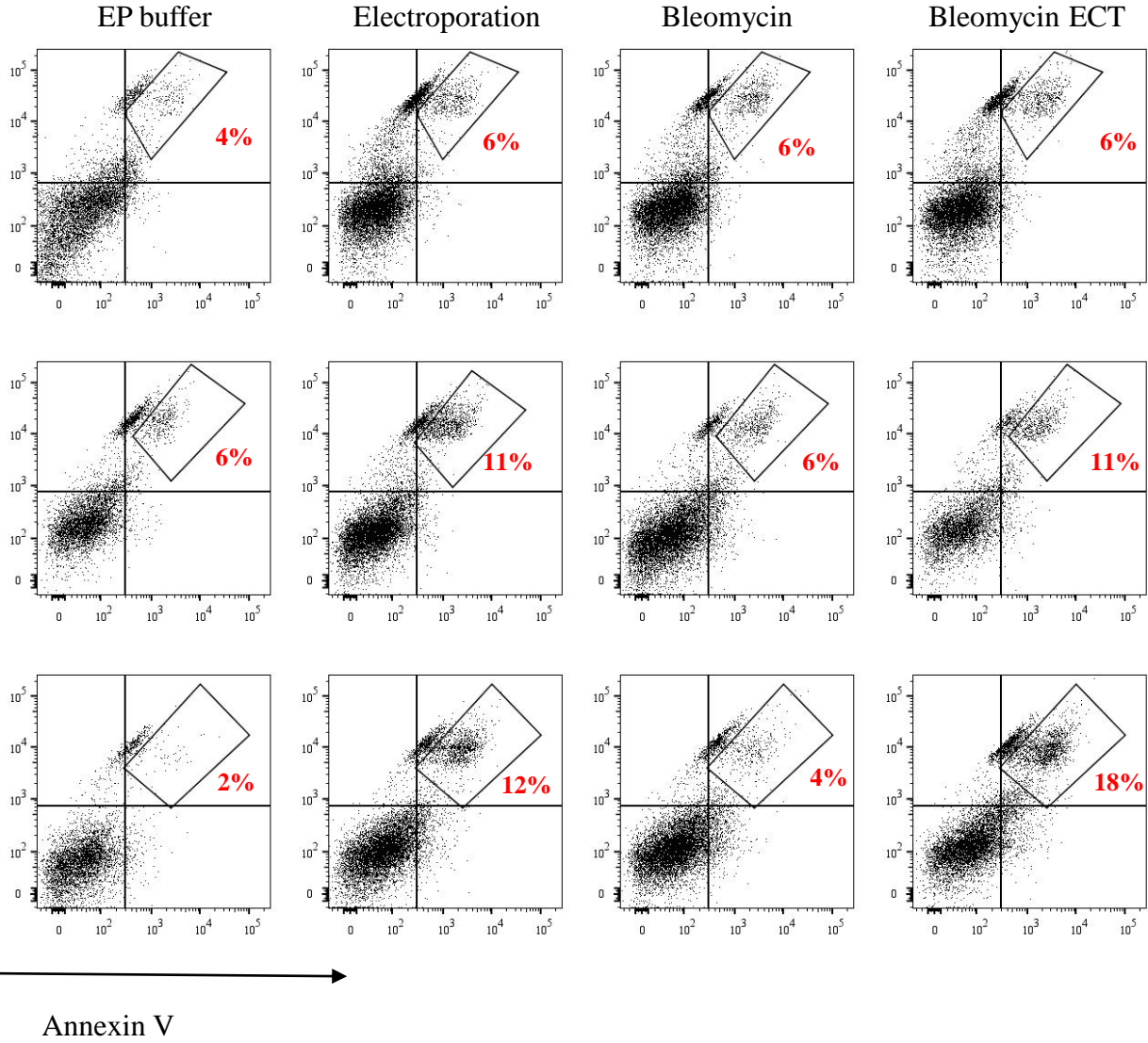

Supplement: Supplementary file 1 [file cancers-11-01177-s001.zip › SD 3.pdf]

Supplementary Figure 4 A

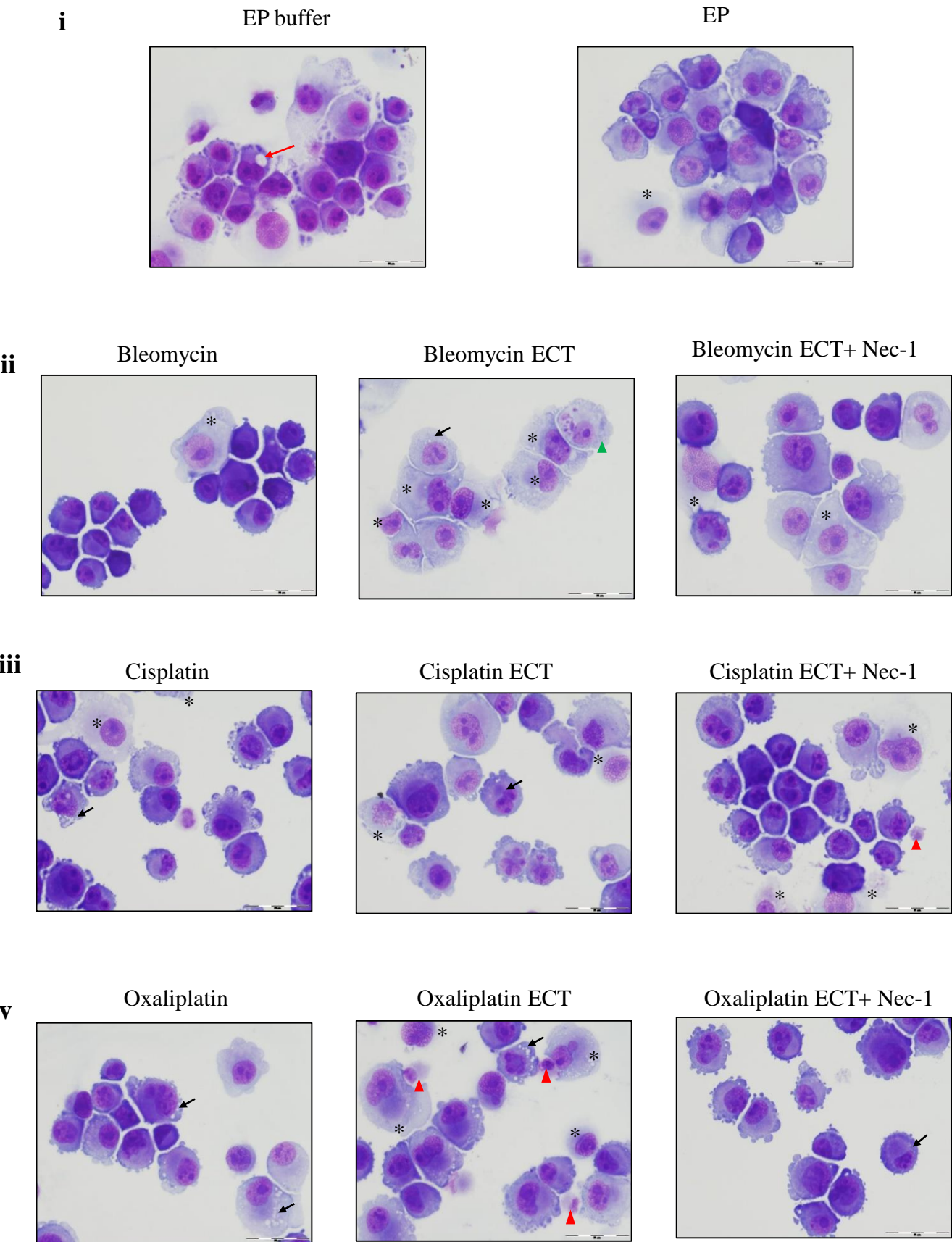

Supplement: Supplementary file 1 [file cancers-11-01177-s001.zip › SD 4A.pdf]

Supplementary Figure 4B

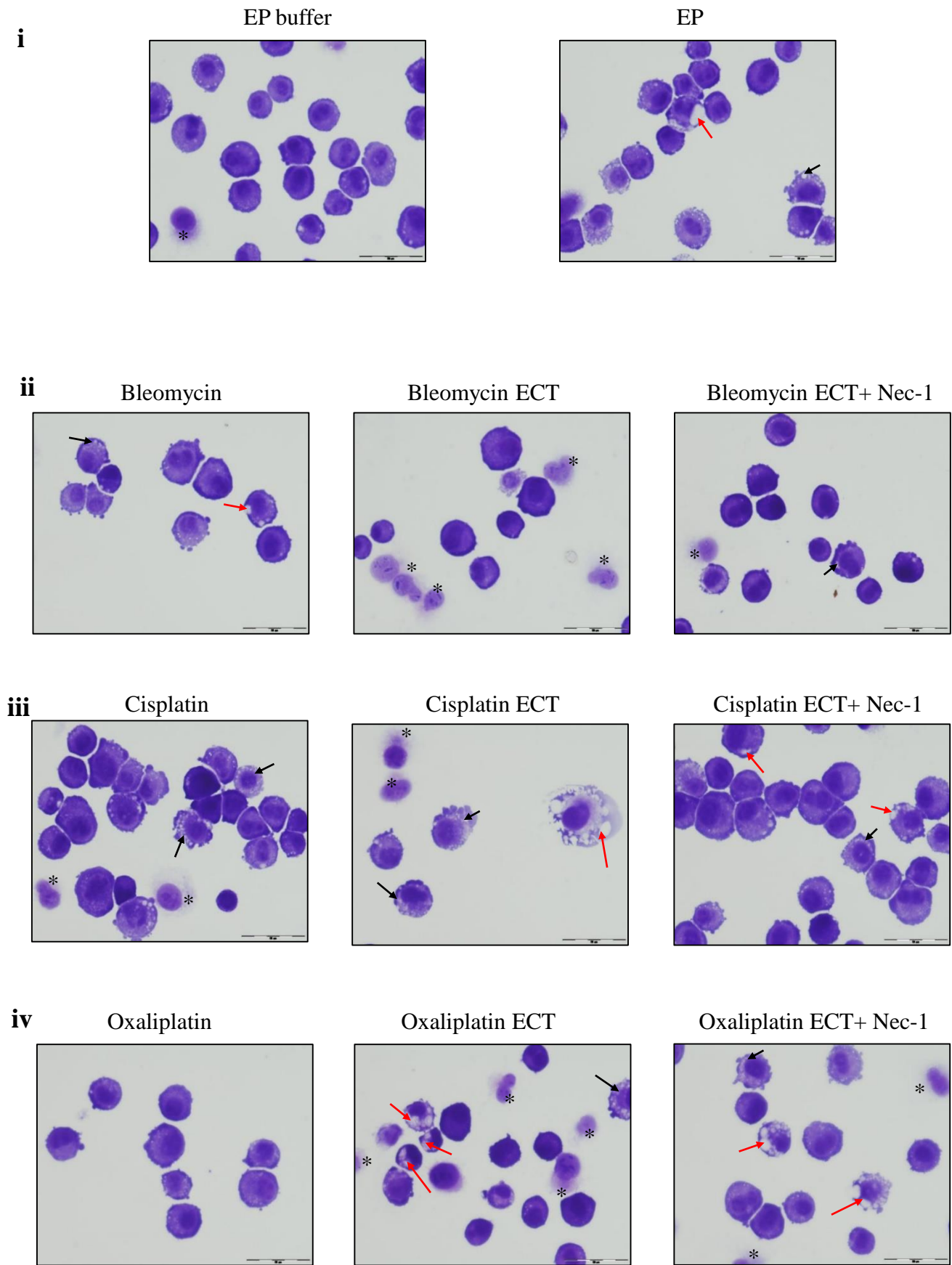

Supplement: Supplementary file 1 [file cancers-11-01177-s001.zip › SD 4B.pdf]

Supplementary Figure 5

A

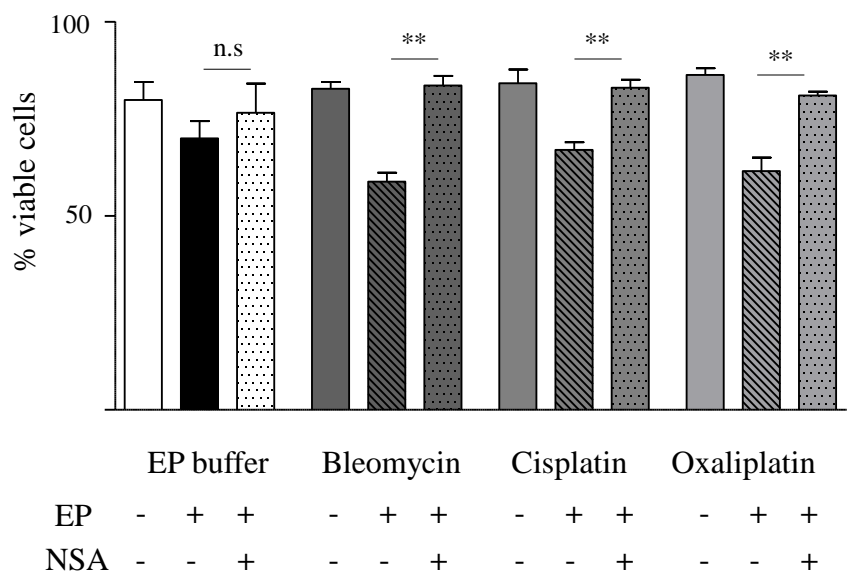

B

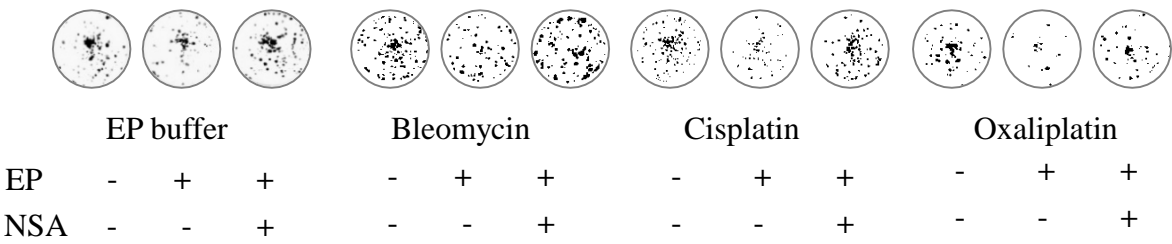

Supplement: Supplementary file 1 [file cancers-11-01177-s001.zip › SD 5.pdf]
